# Comprehensive investigation of *RNF213* nonsynonymous variants associated with intracranial artery stenosis

**DOI:** 10.1038/s41598-020-68888-1

**Published:** 2020-07-20

**Authors:** Hiroki Hongo, Satoru Miyawaki, Hideaki Imai, Masahiro Shimizu, Shinichi Yagi, Jun Mitsui, Hiroyuki Ishiura, Jun Yoshimura, Koichiro Doi, Wei Qu, Yu Teranishi, Atsushi Okano, Hideaki Ono, Hirofumi Nakatomi, Tsuneo Shimizu, Shinichi Morishita, Shoji Tsuji, Nobuhito Saito

**Affiliations:** 1 Department of Neurosurgery, Faculty of Medicine, The University of Tokyo, 7-3-1 Hongo, Bunkyo-ku, Tokyo, 113-8655 Japan; 2 Department of Neurosurgery, Japan Community Healthcare Organization Tokyo Shinjuku Medical Center, Tokyo, Japan; 3 Kanto Neurosurgical Hospital, Kumagaya, Saitama Japan; 4 Department of Molecular Neurology, Graduate School of Medicine, The University of Tokyo, Tokyo, Japan; 5 Department of Neurology, Faculty of Medicine, The University of Tokyo, Tokyo, Japan; 6 Department of Computational Biology and Medical Sciences, Graduate School of Frontier Sciences, The University of Tokyo, Kashiwa, Chiba Japan; 7 School of Bioscience and Biotechnology, Tokyo University of Technology, Tokyo, Japan; 8 Department of Neurosurgery, Fuji Brain Institute and Hospital, Fujinomiya, Shizuoka Japan; 9 International University of Health and Welfare, Narita, Chiba Japan

**Keywords:** Molecular medicine, Risk factors

## Abstract

Intracranial artery stenosis (ICAS) is the most common cause of ischemic stroke worldwide. *RNF213* single nucleotide variant c.14429G > A (p.Arg4810Lys, rs112735431) was recently reported to be associated with ICAS in East Asians. However, the disease susceptibility of other *RNF213* variants has not been clarified. This study comprehensively investigated ICAS-associated *RNF213* variants in a pool of 168 Japanese ICAS patients and 1,194 control subjects. We found 138 nonsynonymous germline variants by target resequencing of all coding exons in *RNF213*. Association study between ICAS patients and control subjects revealed that only p.Arg4810Lys had significant association with ICAS (P = 1.5 × 10^–28^, odds ratio = 29.3, 95% confidence interval 15.31–56.2 [dominant model]). Fourteen of 138 variants were rare variants detected in ICAS patients not harboring p.Arg4810Lys variant. Two of these rare variants (p.Cys118Arg and p.Leu2356Phe) consistent with variants previously reported in moyamoya disease patients characterized by stenosis of intracranial artery and association with *RNF213*, and three rare variants (p.Ser193Gly, p.Val1817Leu, and p.Asp3329Tyr) were found neither in control subjects and Single Nucleotide Polymorphism Database. The present findings may improve our understanding of the genetic background of intracranial artery stenosis.

## Introduction

Intracranial artery stenosis (ICAS) is the most common cause of ischemic stroke worldwide^1–9^ and can result in serious lifelong disabilities^4^. ICAS is also associated with a high risk of recurrent stroke^3,6–8^. The risks of recurrent stroke or death within 1 year and 2 years are 17.5% and 19.5%, respectively, in ICAS patients despite optimum medical management^10^. In addition, ICAS is associated with depression, cognitive deficits, and dementia, and is now increasingly the focus of research.

ICAS is considered to be a major cause of stroke accounting for 30–50% of cases of ischemic stroke in the Asian, African-American, and Hispanic populations, but only about 10% in Caucasians, who are more likely to have extracranial atherosclerotic disease^1–6,8,11–14^. Such racial differences in the prevalence of ICAS have long been a subject of investigations^15^. Various explanations for the difference in the prevalence of ICAS include genetic susceptibility of the intracranial vessels to atherosclerosis, as well as differences in lifestyle and risk factor profiles such as advanced age, hypertension, diabetes, dyslipidemia, cigarette smoking, and metabolic syndrome^4,11^.

We previously identified a genetic variant with a strong association with ICAS^16,17^. This genetic variant is a single base nonsynonymous variant, c.14429G > A (p.Arg4810Lys, rs112735431), which occurs in *ring finger protein 213* (*RNF213*; a gene located in chromosome 17q; based on the National Center for Biotechnology Information Reference sequence NM_001256071.3). This variant is present in approximately 25% of ICAS patients in the Japanese population^16,17^. *RNF213* p.Arg4810Lys also occurs in 1–2% of East Asian populations, such as in Japan, Korea, and China, but is much rarer in European populations^18,19^. Such racial differences in the prevalence of *RNF213* p.Arg4810Lys may be one of the factors responsible for the epidemiological difference in the prevalence of ICAS^18–20^.

*RNF213* encodes a protein of 5,207 amino acids with AAA + ATPase and E3 ligase activities. In vitro and in vivo experiments have suggested that *RNF213* is related to angiogenesis and vascular inflammation, although all of the physiological functions of *RNF213* remain unknown^21^. Clinically, a recent study revealed that *RNF213* p.Arg4810Lys increases the risk of ischemic stroke due to large artery atherosclerosis^22^. Furthermore, *RNF213* p.Arg4810Lys was associated with coronary artery disease and pulmonary hypertension^23–25^. This association between *RNF213* and stenosis of the intracranial, coronary, and other systemic arteries have been extensively investigated recently.

*RNF213* was originally demonstrated as a susceptibility gene for moyamoya disease (MMD), which is characterized by progressive arterial stenosis and occlusion around the circle of Willis^26^. Subsequent study confirmed p.Arg4810Lys as the founder *RNF213* mutation in East Asian patients with MMD^18^. Several studies have searched for additional associated variants other than p.Arg4810Lys in MMD patients. *RNF213* p.Arg4810Lys has a strong association with MMD in the East Asian population^18,26–28^, and has also been detected in MMD patients with several ethnicities such as Indian and Bangladeshi^18,19,26–32^. *RNF213* p.Ala4399Thr was also identified as an associated variant in an association study of the Chinese population^31^. Moreover, other *RNF213* variants have been determined as associated variants in several ethnicities^19,30^. However, additional *RNF213* variants associated with the development of ICAS have been little studied.

The present study searched for additional *RNF213* variants other than p.Arg4810Lys associated with ICAS in the Japanese population by target resequencing of *RNF213* with next generation sequencing, and investigated the detailed genetic background of ICAS involving *RNF213*.

## Materials and methods

### Editorial policies and ethical considerations

This study was approved by the Human Genome, Gene Analysis Research Ethics Committee of the Faculty of Medicine, The University of Tokyo (approval number, 3,516; approval date, September 12, 2011). This study was also approved by the Ethics Committee of Kanto Neurosurgical Hospital (approval date, June 23, 2012). Written informed consent was obtained from all participants or parents/legal guardians for participants under the age of 18. All samples were collected and analyzed in accordance with the relevant guidelines and regulations.

### Study population

One hundred sixty-eight patients with ICAS were recruited for this study from either The University of Tokyo Hospital or Kanto Neurosurgical Hospital between October 2011 and December 2013. All participants were Japanese. The diagnosis of ICAS was based on the findings of magnetic resonance angiography (MRA) (1.5 T or 3 T). MRA images were interpreted by two or more physicians, including at least one radiologist and one neurosurgeon. The ICAS patients included both symptomatic patients and asymptomatic patients who underwent MRA for unrelated symptoms such as headache, vertigo, etc., or medical checkup. Patients diagnosed with non-atherosclerotic vasculopathy, such as dissection, arteritis, or MMD, were excluded. Most of the control group had undergone magnetic resonance imaging which confirmed the absence of cerebrovascular lesions. Clinical data regarding age, sex, hypertension, dyslipidemia, diabetes mellitus, history of cigarette smoking, and systemic atherosclerotic medical history (ischemic heart disease and arteriosclerosis obliterans) were collected via medical records. Hypertension was defined as systolic blood pressure ≥ 140 mmHg or diastolic blood pressure ≥ 90 mmHg, or receiving antihypertensive agents. Dyslipidemia was defined as high-density lipoprotein cholesterol level < 40 mg/dL, low-density lipoprotein cholesterol level ≥ 140 mg/dL, triglyceride level ≥ 150 mg/dL, or receiving lipid-lowering treatment. Diabetes mellitus was defined as HbA1c ≥ 6.5% or being treated with antidiabetic medications. The control group consisted of the whole exome database of 1,194 subjects without any history of brain diseases accumulated at the Department of Genomic Medicine Research Support Center in The University of Tokyo.

### Target resequencing of *RNF213*

Customized SureSelect DNA target enrichment was designed to enrich all the coding regions of *RNF213* (NM_001256071.3) in the ICAS group. The Agilent SureSelect v4 + UTRs or v5 + UTRs kit was used to enrich all genome-wide exome regions in the control group. Sequencing was performed with HiSeq 2,500 (Illumina, Inc., San Diego, CA). Burrows-Wheeler Aligner software^33^ and SAMtools sequence-alignment mapping^34^ was used with the default settings for alignment and variation detection against the human reference genome (National Center for Biotechnology Information build 37 [hg19]).

All identified variants were compared against the Genome Browser (https://genome.ucsc.edu/) database and dbSNP (https://www.ncbi.nlm.nih.gov/snp) and the rs ID of each variant was retrieved, then compared against the Exome Aggregation Consortium (https://exac.broadinstitute.org/) version 0.3.1 data set and the 1,000 genomes database (https://www.internationalgenome.org/) for confirmation of the MAF of each variant. All variants were also compared against the MAF in the Japanese population in the 1,000 genomes database. Variants with MAF ≥ 0.01 in at least one of these databases were regarded as common variants including variants with MAF ≥ 0.01 in Japanese population in the 1,000 genomes database, and variants with MAF < 0.01 in both databases were regarded as rare variants. The impacts of the missense variants on protein function were assessed using three predictors (in-silico analysis): CADD (https://cadd.gs.washington.edu/) (version 1.4), SIFT (https://sift.jcvi.org/) (version 1.1.3), and PolyPhen-2 (https://genetics.bwh.harvard.edu/pph2/) (version 2.2.2) (HumVar), and the impacts of the nonsense variants on protein function were assessed using CADD (https://cadd.gs.washington.edu/) (version 1.4).

We used NM_001256071.3 (NP_001243000.2) as a reference sequence for *RNF213* in this article, which is the major reference sequence for *RNF213* based on the experimentally verified open-reading frame by cDNA cloning^18^.

### Association study

Associations with ICAS of the detected nonsynonymous variants were evaluated by the two-tailed Fisher exact test between ICAS and control subjects with the allele frequency, dominant model, and recessive model: Allele frequency; comparing the total numbers of A and a, dominant model; ‘AA’ versus ‘Aa + aa,’ recessive model; ‘AA + Aa’ versus ‘aa,’ when A and a are the reference allele and alternate allele, respectively.

### Burden test

To test the effect of rare variants on risk increase of disease, a burden test was performed to compare the number of cases with at least one nonsynonymous *RNF213* variant between the ICAS and control groups, since most of the variants detected were rare that the impact of individual variants could not be calculated. We hypothesized that the rarer and more pathogenic variants increase the risk of disease and so characterized variants based on combinations of MAF in Exome Aggregation Consortium and CADD scores^35^, and stratified MAF in Exome Aggregation Consortium into < 0.01, < 0.001, < 0.0001, and < 0.00001, in which MAF was reduced by one tenth stepwise from < 0.01 which defines rare variants to < 0.00001 which defines extremely rare variants (only one or fewer subjects with the variant in the ExAC database), and CADD score into > 0 (all), > 10 (predicted mildly or more damaging), and > 20 (predicted moderately or more damaging), for statistical analysis. CADD grade > 20 was assigned as the highest as the number of variants with > 30 (strongly damaging) was too small for statistical analysis. In this test, frameshift and splice site variants were regarded as variants with deleterious impact on RNF213 function (CADD > 20).

### Review of previously reported *RNF213* variants detected in MMD patients

*RNF213* is known to be associated with MMD, which is characterized by progressive arterial stenosis and occlusion around the circle of Willis^26^. Our previous studies found that *RNF213* p.Arg4810Lys occurs in phenotypes of ICAS and MMD, so both were supposed to have analogous genetic backgrounds. Accordingly, we reviewed previously reported variants detected in MMD patients. The PubMed database was searched for English publications reporting *RNF213* variants detected in MMD patients up to December 2018. We selected studies which performed comprehensive retrieval of *RNF213* variants in MMD patients, and found 14 matched studies that reported *RNF213* variants detected in MMD patients^18,19,26–32,36–40^. One study investigated only exons 41–68 considered to be the genomic hypervariable region of *RNF213*^31^, whereas the other studies comprehensively investigated the entire *RNF213* gene. In this review, we described only information of protein change in each variant because some studies included incomplete descriptions of variants such as those discussing protein changes but not base substitutions. The definitions of common and rare variants were the same as the variants in our series. If information of base changes were not obtained for any variant, we regarded these variants as rare if no common variants were reported corresponding to the protein change.

### Statistical analysis

Continuous variables are described as the median and range. Categorical variables are described using frequencies and percentages and analyzed with the two-tailed Fisher exact test. Association studies used the two-tailed Fisher exact test with allele frequency, dominant model, and recessive model, and multiple testing used the Bonferroni correction for P values calculated with the two-tailed Fisher exact test. For the burden test used logistic regression score test including gender as a covariate and Bonferroni correction. P < 0.05 was considered significant. All data analysis was carried out using JMP Pro version 14.0.0 (SAS Institute, Inc., Cary, NC).

## Results

### Clinical characteristics

The clinical characteristics of the study subjects are shown in Table 1.

**Table 1:** Clinical characteristics of the present subjects.

Characteristics	ICAS (n = 168)
Female sex, n (%)	70 (41.7%)
Age, median, range, y	60, 15–80
Hypertension, n (%)	108 (64.3%)
Diabetes, n (%)	47 (28.0%)
Dyslipidemia, n (%)	57 (33.9%)
Ischemic heart diseases, n (%)	15 (8.9%)
Arteriosclerosis obliterans, n (%)	2 (1.2%)
Smoking, n (%)	44 (26.2%)

ICAS, intracranial artery stenosis.

### Target sequence

Averages of 8.9 million sequence reads were obtained for each sample. Overall, 88.27% of reads were uniquely mapped to the reference genome, with a mean coverage of 2,325× ± 447 in ICAS patients and 64x ± 54 in controls, and 100% and 82.1% of the total target region were covered at 20× in patients and controls, respectively. The sequence data have been submitted to the DNA Data Bank of Japan (DDBJ) database under accession number JGAS00000000175.

### Association study

A total of 138 nonsynonymous variants were confirmed in at least one in our 168 cases in the ICAS series or the whole exome database of 1,194 healthy subjects, consisting of 126 missense variants, 7 frameshift variants, 4 nonsense variants, and 1 splice site variant, which are listed in Supplementary Tables S1 and S2. The association study of the variants showed that p.Arg4810Lys was strongly associated with ICAS (P = 1.5 × 10^–28^, odds ratio [OR] 29.3, 95% confidence interval [CI] 15.31–56.2 [dominant model]) as we found previously^16,17^ (representative results in Table 2 and all results in Supplementary Table S1). No other variants were associated with ICAS.

**Table 2:** Representative results of association study of *RNF213* missense variants in ICAS patients.

	Variant	rs ID	Allele counts (a/A)		Genotype counts (aa/Aa/AA)		OR (95% CI)	P Value
			ICAS	Control	ICAS	Control		
Allele frequency A versus a	p.Arg4810Lys	rs112735431	42/294	13/2,375	1/40/127	0/13/1,181	26.1 (13.8–49.2)	2.1 × 10^–28^*
	p.Asn1045Tyr	–	0/336	0/2,336	0/0/168	26/0/1,168	0	2.0 × 10^–3^
	p.Pro61Leu	rs9913317	20/316	74/2,314	1/18/149	0/74/1,120	1.98 (1.19–3.29)	1.5 × 10^–2^
	p.Gln3082Arg	–	2/334	0/2,388	0/2/166	0/0/1,194	–	1.5 × 10^–2^
	p.Val1195Met	rs10782008	88/248	549/1607	11/66/91	116/549/529	0.73 (0.56–0.94)	1.7 × 10^–2^
Dominant model ‘AA’ versus ‘Aa + aa’	p.Arg4810Lys	rs112735431	42/294	13/2,375	1/40/127	0/13/1,181	29.3 (15.3–56.2)	1.5 × 10^–28^*
	p.Gln3082Arg	–	2/334	0/2,388	0/2/166	0/0/1,194	–	1.5 × 10^–2^
	p.Val1195Met	rs10782008	88/248	549/1607	11/66/91	116/549/529	0.67 (0.49–0.93)	2.0 × 10^–2^
	p.Pro61Leu	rs9913317	20/316	74/2,314	1/18/149	0/74/1,120	1.93 (1.13–3.29)	2.1 × 10^–2^
	p.Ala1041Thr	rs61359568	6/330	80/2,306	1/4/163	1/80/1,113	0.42 (0.17–1.06)	2.6 × 10^–2^
Recessive model ‘AA + Aa’ versus ‘aa’	p.Asn1045Tyr	–	0/336	0/2,336	0/0/168	26/0/1,168	0	6.4 × 10^–2^
	p.Met270Thr	rs17857135	35/301	189/2,193	2/31/135	3/189/1,002	4.78 (0.79–28.8)	1.2 × 10^–1^
	p.Arg4810Lys	rs112735431	42/294	13/2,375	1/40/127	0/13/1,181	–	1.2 × 10^–1^
	p.Pro61Leu	rs9913317	20/316	74/2,314	1/18/149	0/74/1,120	–	1.2 × 10^–1^
	p.Asp2554Glu	rs138516230	5/331	29/2,359	1/3/164	0/29/1,165	–	1.2 × 10^–1^

*Statistically significant (Bonferroni corrected significance level is 3.6 × 10^–4^ [0.05/138]).

CI, confidence interval; OR, odds ratio.

### Burden test

Results of the burden tests are shown in Table 3. Variants with minor allele frequency (MAF) < 0.01 and Combined Annotation Dependent Depletion (CADD) > 0, variants with MAF < 0.01 and CADD > 10, variants with MAF < 0.001 and CADD > 0, and variants with MAF < 0.001 and CADD > 10 were associated with increased risk of ICAS (OR 1.97, 95% CI 1.42–2.73; OR 2.64, 95% CI 1.87–3.73; OR 2.39, 95% CI 1.68–3.41; and OR 4.56, 95% CI 3.10–6.73, respectively). However, p.Arg4810Lys (MAF 0.0004 and CADD 12.8) included in these groups of variants was thought to have greatly influenced the results of these categories. Restudy after excluding subjects with p.Arg4810Lys (Supplementary Table S3) found no category showing significant differences between the ICAS and control groups. Consequently, the burden test found no significant association for variants other than p.Arg4810Lys.

**Table 3:** Results of burden tests for nonsynonymous variants detected in our subjects.

	N-variants in burden test	Cases with at least one variant (total 168)	Controls with at least one variant (total 1,194)	OR (95% CI)	P Value
**Any-MAF**	56				
CADD > 0	56	168	1,189	–	0.99
CADD > 10	25	168	1,189	–	0.99
CADD > 20	7	5	49	0.74 (0.29–1.88)	0.52
**MAF < 0.01**	42				
CADD > 0	42	81	382	2.01 (1.45–2.80)	2.8 × 10^–5^*
CADD > 10	22	63	220	2.71 (1.92–3.84)	1.7 × 10^–8^*
CADD > 20	7	5	45	0.81 (0.31–2.07)	0.65
**MAF < 0.001**	36				
CADD > 0	36	57	210	2.54 (1.78–3.62)	3.0 × 10^–7^*
CADD > 10	21	50	101	4.92 (3.32–7.31)	2.7 × 10^–15^*
CADD > 20	7	5	45	0.81 (0.31–2.07)	0.65
**MAF < 0.0001**	29				
CADD > 0	29	19	123	1.16 (0.69–1.93)	0.58
CADD > 10	19	14	80	1.30 (0.72–2.35)	0.39
CADD > 20	7	5	43	0.84 (0.33–2.15)	0.71
**MAF < 0.00001**	20				
CADD > 0	20	14	84	1.26 (0.69–2.27)	0.45
CADD > 10	15	11	60	1.38 (0.71–2.68)	0.35
CADD > 20	6	4	33	0.89 (0.31–2.55)	0.82

*Statistically significant (Bonferroni corrected significance level is 3.3 × 10^–3^ [0.05/15]).

CADD, combined annotation dependent depletion; MAF, minor allele frequency.

### Investigation of rare variants in ICAS patients; comparison with variants previously reported in MMD patients, and search in control subjects and single nucleotide polymorphism database (dbSNP)

**Figure 1:**
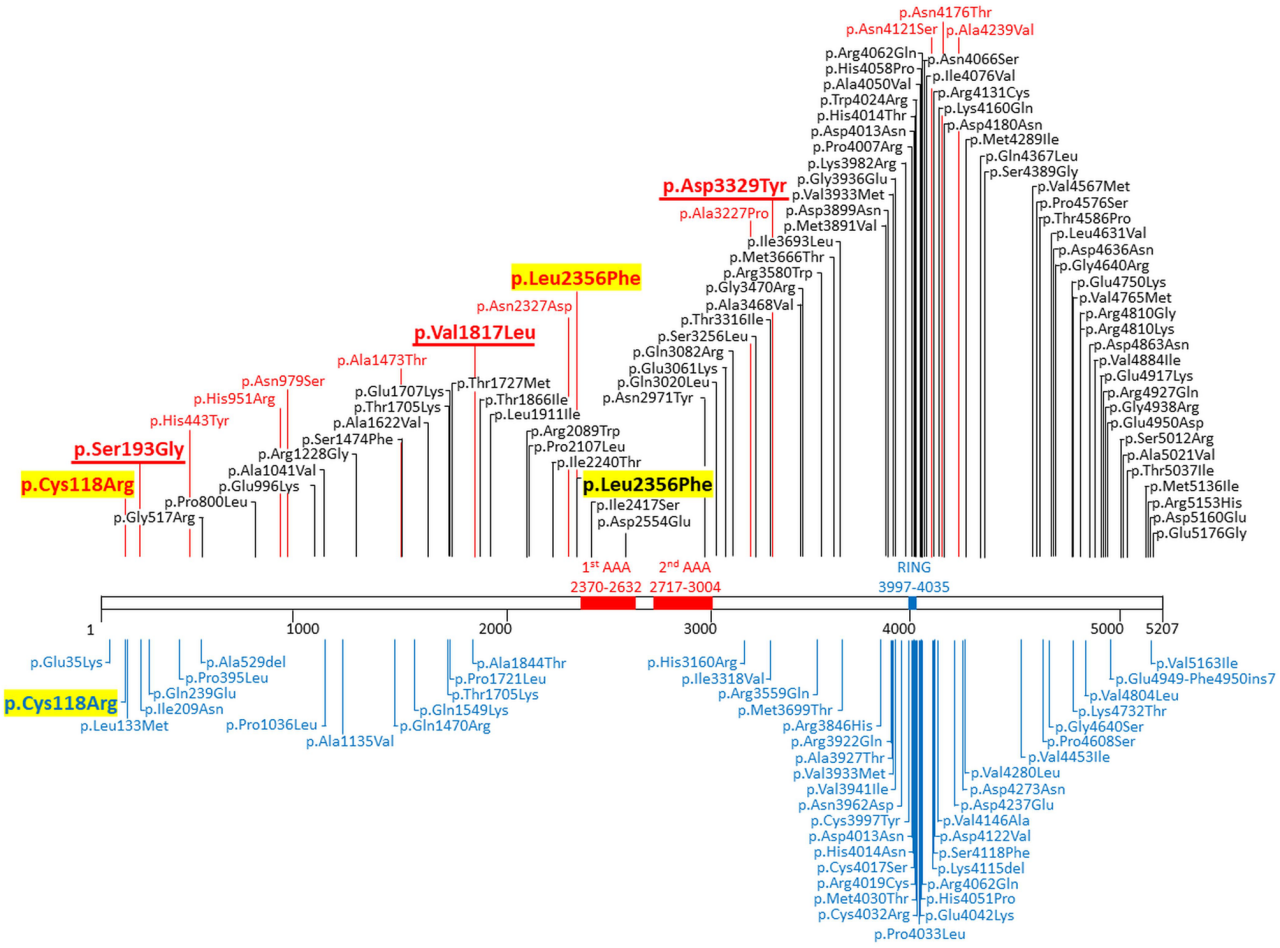
*RNF213* nonsynonymous rare variants found in our intracranial artery stenosis patients without p.Arg4810Lys and moyamoya disease (MMD) patients in previously reported studies. Variants found in the present study are marked in red, and previously reported *RNF213* variants in MMD patients in the Asian population and non-Asian population are marked in black and blue, respectively. p.Cys118Arg and p.Leu2356Phe corresponded with two of the variants in MMD patients. p.Ser193Gly, p.Val1817Leu, and p.Asp3329Tyr were variants found in neither control subjects nor Single Nucleotide Polymorphism Database.

Fourteen of the 138 nonsynonymous variants in our series were rare variants detected in ICAS patients not harboring p.Arg4810Lys (Table 4), which were regarded as the candidate rare variants associated with ICAS other than p.Arg4810Lys. Review of *RNF213* variants in MMD patients identified one common variant (p.Ala4399Thr) with statistically verified association with MMD^31^ and 117 rare variants (Supplementary Table S4). Seventy-two rare variants occurred in Asian MMD patients, and 49 in non-Asian patients (4 variants occurred in both Asian and non-Asian patients). The *RNF213* rare variants in MMD patients and variants detected in our ICAS series are included in the schema of *RNF213* (Fig. 1). p.Cys118Arg and p.Leu2356Phe, which were identified in our ICAS patients without p.Arg4810Lys, were also found among the *RNF213* variants in MMD patients. Three of the other 12 rare variants, p.Ser193Gly, p.Val1817Leu, and p.Asp3329Tyr, were found in neither control subjects nor dbSNP.

**Table 4:** Rare variants detected in ICAS patients without p.Arg4810Lys.

cDNA (NM_001256071.3)	Protein (NP_001243000.2)	rs ID	ICAS aa/Aa/AA	Control aa/Aa/AA	MAF in ExAC	MAF in 1,000 Genomes	Found in dbSNP	Previously reported in MMD patients	CADD	SIFT	PolyPhen-2
c.352 T > C	p.Cys118Arg	rs201620985	0/1/167	0/0/1,194	0.0026	0.0002	Yes	Yes	1.792	Tolerated	Benign
c.577A > G	p.Ser193Gly	–	0/1/167	0/0/1,194	0	0	No	No	0.498	Tolerated	Benign
c.1327C > T	p.His443Tyr	rs199729731	0/1/167	0/2/1,192	0	0.0002	Yes	No	0.172	Tolerated	Benign
c.2852A > G	p.His951Arg	rs746475303	0/1/167	0/3/1,191	0	0	Yes	No	11.62	Tolerated	Benign
c.2936A > G	p.Asn979Ser	rs1049581142	0/1/167	0/1/1,193	0	0	Yes	No	2.096	Tolerated	Benign
c.4417G > A	p.Ala1473Thr	rs1428500978	0/1/167	0/2/1,192	0	0	Yes	No	14.96	D*	Possibly D*
c.5449G > T	p.Val1817Leu	–	0/1/167	0/0/1,194	0	0	No	No	10.08	D*	Possibly D*
c.6979A > G	p.Asn2327Asp	rs138044665	0/1/167	0/0/1,194	0.0009	0.0016	Yes	No	4.35	Tolerated	Benign
c.7066C > T	p.Leu2356Phe	rs200724769	0/1/167	0/3/1,191	0.00008	0	Yes	Yes	14.16	D*	Benign
c.9679G > C	p.Ala3227Pro	rs763302578	0/1/167	0/4/1,190	0	0	Yes	No	22.8	D*	Probably D*
c.9985G > T	p.Asp3329Tyr	–	0/1/167	0/0/1,194	0	0	No	No	24.6	D*	Probably D*
c.12362A > G	p.Asn4121Ser	rs143828863	0/1/167	0/1/1,193	0.00008	0	Yes	No	19.39	Tolerated	Benign
c.12527A > G	p.Asn4176Ser	rs527844265	0/1/167	0/1/1,193	0	0	Yes	No	15.28	D*	Benign
c.12716C > T	p.Ala4239Val	–	0/2/166	0/1/1,193	0	0	No	No	18.2	Tolerated	Probably D*

Exons and amino acid positions are provided according to the NM_001256071.3 isoform. A, reference allele; a, alternate allele; D*, damaging; dbSNP, single nucleotide polymorphism database; ExAC, exome aggregation consortium; 1,000 Genomes, 1,000 genomes project; –, novel variant.

### Discussion

The present study identified 138 nonsynonymous *RNF213* variants in our series of 168 ICAS patients and 1,194 healthy subjects. The association study found only one variant, *RNF213* p.Arg4810Lys, with significant difference between the ICAS and control groups. This result corresponds to our previous findings^16,17^ and reconfirmed the importance of *RNF213* p.Arg4810Lys in Japanese ICAS patients. Other rare variants detected in ICAS patients not harboring p.Arg4810Lys included p.Cys118Arg and p.Leu2356Phe, which were previously detected in MMD patients, and p.Ser193Gly, p.Val1817Leu, and p.Asp3329Tyr, which were identified in neither control subjects nor dbSNP, so were considered as important candidate variants indicating susceptibility for intracranial artery stenosis. The present study comprehensively investigated all coding exons on *RNF213* in Japanese ICAS patients for the detection of ICAS-associated *RNF213* variants. Further investigations may identify the etiology of intracranial artery stenosis including ICAS and MMD.

*RNF213* encodes a protein with 5,207 amino acids harboring a RING (really interesting new gene) finger motif and an AAA (adenosine triphosphatase associated with various cellular activities) domain, indicating the presence of both E3 ubiquitin ligase activity and energy-dependent unfoldase^18^. Several studies have suggested that *RNF213* is associated with intracranial arterial wall construction. Knockdown of *RNF213* in zebrafish caused irregular wall formation in the trunk arteries and abnormal sprouting vessels^18^. *RNF213*-knockout mice showed thinning of the intima and media layer and reduced cerebral blood flow under cerebral hypoperfusion^41^. Several in vitro studies have also shown *RNF213* variants cause changes in angiogenic activity^36,42,43^. All of these results suggest that *RNF213* is involved in the intracranial artery remodeling process.

The present study showed a strong association between *RNF213* p.Arg4810Lys and ICAS, which extended our previous findings of a relationship between intracranial artery stenosis/occlusion and *RNF213* p.Arg4810Lys. *RNF213* was first identified as a susceptibility gene for MMD, but clearly *RNF213* p.Arg4810Lys is involved in various patterns of intracranial artery stenotic phenotypes including ICAS, which suggests that *RNF213* p.Arg4810Lys is involved in some complex mechanism affecting intracranial artery remodeling. A recent study demonstrated the association of *RNF213* with intracranial aneurysms in French-Canadian patients, which are characterized by thin tunica media and internal elastic lamina in contrast to MMD and other intracranial artery stenotic diseases^44^. Therefore, *RNF213* may be involved in intracranial vascular pathophysiologies in addition to artery stenosis diseases. Furthermore, other studies recently showed that *RNF213* p.Arg4810Lys is associated with coronary artery disease and pulmonary hypertension^23–25^. Consequently, *RNF213* may also be involved in systemic artery diseases other than intracranial artery disease.

Two *RNF213* rare variants, p.Cys118Arg and p.Leu2356Phe, were identified in our ICAS patients without *RNF213* p.Arg4810Lys, and previously in Caucasian and Japanese MMD patients, respectively^27,37^. These independent findings of *RNF213* p.Cys118Arg and p.Leu2356Phe in two series of intracranial artery stenosis diseases suggest that these variants are important candidates for intracranial artery stenosis. Accordingly, *RNF213* p.Cys118Arg and p.Leu2356Phe may be associated with various intracranial artery stenotic phenotypes including ICAS and MMD. Even within the same *RNF213* gene, the loci of variants are related to different subtypes of MMD; p.Arg4810Lys is associated with ischemic type MMD, whereas p.Ala4399Thr is associated with hemorrhagic type MMD^31^. Similarly, *RNF213* p.Cys118Arg and p.Leu2356Phe may cause phenotypes closer to ICAS rather than MMD. In addition, three rare variants, p.Ser193Gly, p.Val1817Leu, and p.Asp3329Tyr, identified in our ICAS patients without *RNF213* p.Arg4810Lys were absent from both the control subjects and dbSNP, and so were considered as candidate variants associated with ICAS. Further study may reveal the detailed functions of these rare variants.

Additional *RNF213* variants associated with MMD have been reported in several ethnicities, other than p.Arg4810Lys which is strongly associated with East Asian MMD patients and is thought to be a founder mutation in this population^19,30^. The distributions of such variants are also important. Most MMD-associated *RNF213* variants, including the possible founder variant p.Arg4810Lys, are located in the C terminus of the *RNF213* protein including the ring finger domain in both East Asians and Caucasians^18,19,21,26,37^. The present study also showed accumulation of variants in MMD patients in the C terminus. p.Arg4810Lys and some non-p.Arg4810Lys variants located in the C terminus are known to affect angiogenic activity^36^. In contrast, study of the association between aneurysms in French-Canadian patients and *RNF213* demonstrated that the deleterious variant in AAA + motifs contributed to aneurysm formation based on the measurement of adenosine triphosphatase activity^44^. These findings may indicate that each phenotype has a discrete distribution of associated variants along the entire length of *RNF213*.

The present study showed that variants in ICAS patients were not clustered in the C terminus but dispersed between the N terminus and the region around the AAA domain, unlike variants reported in MMD patients. These findings may indicate that *RNF213* variants in patients with ICAS have different distributions from those in MMD patients. Alternatively, the variants not located in the C terminus may have mild impacts on the intracranial arteries and manifest as ICAS instead of MMD. The impact of each variant on ICAS pathogenicity should be clarified.

The present study has limitations with the scale of analysis of variants having minor impact on disease susceptibility or low frequency, and the numbers of patients and control subjects were insufficient for detection of distinct ICAS-associated rare variants. However, this study successfully detected several variants as candidate rare variants causing intracranial artery stenosis based on the comparison with previously reported studies of MMD patients, control subjects, and dbSNP. More definite association of these rare variants with ICAS may be verified with a larger replication study in the future.

## Conclusions

Comprehensive investigation of *RNF213* variants revealed that *RNF213* p.Arg4810Lys is strongly associated with ICAS patients in the Japanese population, and identified *RNF213* p.Cys118Arg, p.Ser193Gly, p.Val1817Leu, p.Leu2356Phe, and p.Asp3329Tyr as candidate variants associated with intracranial artery stenosis.

## Data availability

The sequence data of our ICAS patients have been submitted to the DDBJ database under Accession Number JGAS00000000175. The datasets of control subjects analyzed during the current study are available from the corresponding author on reasonable request.

## Supplementary information

Supplementary file
