# Supplementary material for: Comprehensive investigation of RNF213 nonsynonymous variants associated with intracranial artery stenosis
Source: Sci Rep. 2020 Jul 20;10:11942. doi: 10.1038/s41598-020-68888-1 (PMC7371676; doi:10.1038/s41598-020-68888-1)
Supplement: Supplementary file 1 — Supplementary file [file 41598_2020_68888_MOESM1_ESM.pdf]

Supplementary Information:

Comprehensive Investigation of *RNF213* Nonsynonymous Variants Associated with Intracranial Artery Stenosis

Hiroki Hongo[1], Satoru Miyawaki[1,*], Hideaki Imai[2], Masahiro Shimizu[3], Shinichi Yagi[3], Jun Mitsui[4], Hiroyuki Ishiura[5], Jun Yoshimura[6], Koichiro Doi[6,7], Wei Qu[6], Yu Teranishi[1], Atsushi Okano[1], Hideaki Ono[8], Hirofumi Nakatomi[1], Tsuneo Shimizu[3], Shinichi Morishita[6], Shoji Tsuji[4,9], and Nobuhito Saito[1]

[1]Department of Neurosurgery, Faculty of Medicine, The University of Tokyo, Tokyo, Japan

[2]Department of Neurosurgery, Japan Community Healthcare Organization Tokyo Shinjuku Medical Center, Tokyo, Japan

[3]Kanto Neurosurgical Hospital, Kumagaya, Saitama, Japan

[4]Department of Molecular Neurology, Graduate School of Medicine, The University of Tokyo, Tokyo, Japan

[5]Department of Neurology, Faculty of Medicine, The University of Tokyo, Tokyo, Japan

[6]Department of Computational Biology and Medical Sciences, Graduate School of Frontier Sciences, The University of Tokyo, Kashiwa, Chiba, Japan

[7]School of Bioscience and Biotechnology, Tokyo University of Technology, Tokyo, Japan

[8]Department of Neurosurgery, Fuji Brain Institute and Hospital, Fujinomiya, Shizuoka, Japan

[9]International University of Health and Welfare, Narita, Chiba, Japan

*Correspondence: Satoru Miyawaki, MD, PhD, Department of Neurosurgery, Faculty of Medicine, The University of Tokyo, 7-3-1 Hongo, Bunkyo-ku, Tokyo 113-8655, Japan

Tel.: +81-3-5800-8853; Fax: +81-3-5800-8655

E-mail address: miyawaki-tky@umin.ac.jp

Supplementary Table S1. Nonsynonymous variants of *RNF213* in ICAS patients and control subjects with the results of association studies

cDNA (NM_001256071.3)	Protein (NP_001243000.2)	rs ID	Allele counts (a/A)		Genotype counts (aa/Aa/AA)		p values of association studies		
			ICAS	Control	ICAS	Control	Allele frequency A vs. a	Dominant model 'AA' vs. 'Aa + aa'	Recessive model 'AA + Aa' vs. 'aa'
c.103G>A	p.Glu35Lys	rs113614776	0/336	1/2387	0/0/168	0/1/1193	1	1	-
c.182C>T	p.Pro61Leu	rs9913317	20/316	74/2314	1/18/149	0/74/1120	0.02	0.02	0.12
c.352T>C	p.Cys118Arg	rs201620985	1/335	0/2388	0/1/167	0/0/1194	0.12	0.12	-
c.355C>T	p.His119Tyr	rs775609914	0/336	1/2387	0/0/168	0/1/1193	1	1	-
c.577A>G	p.Ser193Gly	-	1/335	0/2388	0/1/167	0/0/1194	0.12	0.12	-
c.752_753insT	p.Glu252*	-	0/336	1/2387	0/0/168	0/1/1193	1	1	-
c.757C>T	p.Pro253Ser	rs140369116	2/334	2/2386	0/2/166	0/2/1192	0.08	0.08	-
c.809T>C	p.Met270Thr	rs17857135	35/301	189/2193	2/31/135	3/189/1002	0.17	0.27	0.12
c.962T>C	p.Met321Thr	rs17853989	35/301	177/2203	2/31/135	4/177/1013	0.11	0.14	0.16
c.968C>G	p.Ala323Gly	-	0/336	1/2387	0/0/168	0/1/1193	1	1	-
c.1035G>C	p.Lys345Asn	-	0/336	1/2387	0/0/168	0/1/1193	1	1	-
c.1045G>A	p.Ala349Thr	-	0/336	1/2387	0/0/168	0/1/1193	1	1	-
c.1052C>T	p.Ala351Val	rs148593553	0/336	2/2386	0/0/168	0/2/1192	1	1	-
c.1207A>G	p.Arg403Gly	rs773721359	0/336	1/2387	0/0/168	0/1/1193	1	1	-
c.1271G>A	p.Arg424Lys	-	1/335	0/2388	0/1/167	0/0/1194	0.12	0.12	-

c.1327C>T	p.His443Tyr	rs199729731	1/335	2/2386	0/1/167	0/2/1192	0.33	0.33	-
c.1762A>G	p.Arg588Gly	rs1390431807	0/336	1/2387	0/0/168	0/1/1193	1	1	-
c.2186C>T	p.Pro729Leu	rs72849841	2/334	51/2337	0/2/166	0/51/1143	0.06	0.05	-
c.2852A>G	p.His951Arg	rs746475303	1/335	3/2385	0/1/167	0/3/1191	0.41	0.41	-
c.2867C>T	p.Ser956Leu	rs773701444	0/336	1/2387	0/0/168	0/1/1193	1	1	-
c.2936A>G	p.Asn979Ser	rs1049581142	1/335	1/2387	0/1/167	0/1/1193	0.23	0.23	-
c.3121G>A	p.Ala1041Thr	rs61359568	6/330	80/2306	1/4/163	1/80/1113	0.03	0.06	1
c.3133G>T	p.Asp1045Tyr	-	0/336	0/2336	0/0/168	26/0/1168	0.002	0.06	0.06
c.3286A>G	p.Ile1047Val	-	0/336	1/2387	0/0/168	0/1/1193	1	1	-
c.3191G>A	p.Cys1064Tyr	rs1437417566	0/336	4/2384	0/0/168	0/4/1190	1	1	-
c.3212T>C	p.Leu1071Pro	-	0/336	1/2387	0/0/168	0/1/1193	1	1	-
c.3397C>A	p.Gln1133*	-	71/265	423/1849	0/0/168	3/0/1191	1	1	1
c.3397C>T	p.Gln1133Lys	rs8082521	0/336	0/2382	8/55/105	58/423/713	0.58	0.5	1
c.3512T>C	p.Ile1171Thr	-	0/336	1/2387	0/0/168	0/1/1193	1	1	-
c.3583G>A	p.Val1195Met	rs10782008	88/248	549/1607	11/66/91	116/549/529	0.02	0.02	0.2
c.3701A>G	p.Gln1234Arg	rs116722283	0/336	1/2387	0/0/168	0/1/1193	1	1	-
c.3814G>C	p.Glu1272Gln	rs9913636	82/254	513/1713	10/62/96	81/513/600	0.15	0.1	0.87
c.3992A>G	p.Asp1331Gly	rs8074015	104/232	565/1551	16/72/80	136/565/493	0.14	0.13	0.6
c.3992A>T	p.Asp1331Val	-	0/336	0/2384	0/0/168	2/0/1192	1	1	1
c.4031A>G	p.Lys1344Arg	-	0/336	1/2387	0/0/168	0/1/1193	1	1	-
c.4274A>G	p.Lys1425Arg	-	0/336	1/2387	0/0/168	0/1/1193	1	1	-
c.4316A>G	p.Asn1439Ser	rs761802191	0/336	1/2387	0/0/168	0/1/1193	1	1	-

c.4336G>C	p.Asp1446His	-	0/336	1/2387	0/0/168	0/1/1193	1	1	-
c.4417G>A	p.Ala1473Thr	rs1428500978	1/335	2/2386	0/1/167	0/2/1192	0.33	0.33	-
c.4626A>C	p.Gln1542His	-	0/336	1/2387	0/0/168	0/1/1193	1	1	-
c.4691A>G	p.His1564Arg	rs1568090106	0/336	1/2387	0/0/168	0/1/1193	1	1	-
c.4865C>A	p.Ala1622Asp	rs534665632	0/336	1/2387	0/0/168	0/1/1193	1	1	-
c.5132A>G	p.Tyr1711Cys	-	0/336	2/2386	0/0/168	0/2/1192	1	1	-
c.5221G>A	p.Val1741Ile	rs1392164744	0/336	1/2387	0/0/168	0/1/1193	1	1	-
c.5267G>A	p.Arg1756His	rs772938055	0/336	2/2386	0/0/168	0/2/1192	1	1	-
c.5449G>T	p.Val1817Leu	-	1/335	0/2388	0/1/167	0/0/1194	0.12	0.12	-
c.5577C>G	p.Tyr1859*	-	0/336	2/2386	0/0/168	0/2/1192	1	1	-
c.5597C>T	p.Thr1866Ile	rs546687179	0/336	6/2382	0/0/168	0/6/1188	1	1	-
c.5795G>A	p.Gly1932Glu	-	0/336	1/2387	0/0/168	0/1/1193	1	1	-
c.5909C>T	p.Pro1970Leu	rs768990872	0/336	1/2387	0/0/168	0/1/1193	1	1	-
c.5972C>T	p.Ser1991Leu	rs1164127473	0/336	1/2387	0/0/168	0/1/1193	1	1	-
c.6019G>A	p.Asp2007Asn	rs369925564	1/335	3/2385	0/1/167	0/3/1191	0.41	0.41	-
c.6034C>T	p.Gln2012*	-	0/336	1/2387	0/0/168	0/1/1193	1	1	-
c.6202G>A	p.Val2068Met	-	0/336	1/2387	0/0/168	0/1/1193	1	1	-
c.6320C>T	p.Pro2107Leu	rs756213942	1/335	1/2387	0/1/167	0/1/1193	0.23	0.23	-
c.6575G>A	p.Gly2192Asp	rs755712045	1/335	0/2388	0/1/167	0/0/1194	0.12	0.12	-
c.6611A>G	p.His2204Arg	rs774152938	0/336	1/2387	0/0/168	0/1/1193	1	1	-
c.6659G>A	p.Arg2220Gln	rs764839860	0/336	1/2387	0/0/168	0/1/1193	1	1	-
c.6802C>T	p.His2268Tyr	rs764823009	0/336	1/2387	0/0/168	0/1/1193	1	1	-

c.6979A>G	p.Asn2327Asp	rs138044665	1/335	0/2388	0/1/167	0/0/1194	0.12	0.12	-
c.7001G>A	p.Ser2334Asn	rs9674961	98/238	531/1627	15/68/85	115/531/548	0.35	0.28	0.89
c.7066C>T	p.Leu2356Phe	rs200724769	1/335	3/2385	0/1/167	0/3/1191	0.41	0.41	-
c.7232G>A	p.Arg2411Gln	rs768216512	1/335	2/2386	0/1/167	0/2/1192	0.33	0.33	-
c.7250T>G	p.Ile2417Ser	rs181965032	0/336	3/2385	0/0/168	0/3/1191	1	1	-
c.7319G>A	p.Gly2440Asp	rs761027115	0/336	1/2387	0/0/168	0/1/1193	1	1	-
c.7348A>G	p.Lys2450Glu	rs1299071699	0/336	1/2387	0/0/168	0/1/1193	1	1	-
c.7662C>A	p.Asp2554Glu	rs138516230	5/331	29/2359	1/3/164	0/29/1165	0.6	1	0.12
c.8020A>G	p.Asn2674Asp	-	0/336	1/2387	0/0/168	0/1/1193	1	1	-
c.8217G>T	p.Arg2739Ser	-	0/336	1/2387	0/0/168	0/1/1193	1	1	-
c.8389C>A	p.Arg2797Ser	rs776731965	0/336	1/2387	0/0/168	0/1/1193	1	1	-
c.8983G>A	p.Ala2995Thr	rs374068975	0/336	1/2387	0/0/168	0/1/1193	1	1	-
c.9013G>A	p.Glu3005Lys	rs147076172	0/336	1/2387	0/0/168	0/1/1193	1	1	-
c.9022T>C	p.Cys3008Arg	rs61600413	15/321	58/2330	0/15/153	0/58/1136	0.04	0.04	-
c.9245A>G	p.Gln3082Arg	-	2/334	0/2388	0/2/166	0/0/1194	0.02	0.02	-
c.9404C>T	p.Thr3135Ile	-	0/336	2/2386	0/0/168	0/2/1192	1	1	-
c.9422G>A	p.Arg3141Gln	-	0/336	1/2387	0/0/168	0/1/1193	1	1	-
c.9439C>T	p.Arg3147Cys	rs781176178	0/336	1/2387	0/0/168	0/1/1193	1	1	-
c.9542A>G	p.Glu3181Gly	-	0/336	1/2387	0/0/168	0/1/1193	1	1	-
c.9679G>C	p.Ala3227Pro	rs776295572	1/335	4/2384	0/1/167	0/4/1190	0.48	0.48	-
c.9767C>T	p.Ser3256Leu	rs529113475	0/336	1/2387	0/0/168	0/1/1193	1	1	-
c.9806G>C	p.Arg3269Pro	rs377303933	0/336	1/2387	0/0/168	0/1/1193	1	1	-

c.9909C>A	p.His3303Gln	-	0/336	1/2387	0/0/168	0/1/1193	1	1	-
c.9985G>T	p.Asp3329Tyr	-	1/335	0/2388	0/1/167	0/0/1194	0.12	0.12	-
c.10403C>T	p.Ala3468Val	rs142798005	2/334	21/2367	0/2/166	0/21/1173	1	1	-
c.10617G>C	p.Glu3490Asp	rs7216493	0/336	0/2380	0/0/168	4/0/1190	0.61	1	1
c.10491_10492insGGAGGTGGCAGAGG	p.Glu3498Glyfs*44	-	0/336	1/2387	0/0/168	0/1/1193	1	1	-
c.10510G>A	p.Glu3504Lys	rs751952931	0/336	1/2387	0/0/168	0/1/1193	1	1	-
c.10526_10527insCA	p.Glu3510Lysfs*28	-	0/336	1/2387	0/0/168	0/1/1193	1	1	-
c.10532G>A	p.Ser3511Asn	rs749427375	0/336	1/2387	0/0/168	0/1/1193	1	1	-
c.10579G>A	p.Val3527Met	rs547259103	0/336	2/2386	0/0/168	0/2/1192	1	1	-
c.10663A>G	p.Thr3555Ala	-	0/336	2/2386	0/0/168	0/2/1192	1	1	-
c.10675C>T	p.Arg3559Trp	rs368365560	0/336	2/2386	0/0/168	0/2/1192	1	1	-
c.10684G>C	p.Val3562Leu	-	0/336	1/2387	0/0/168	0/1/1193	1	1	-
c.10727-2A>G	-	-	0/336	1/2387	0/0/168	0/1/1193	1	1	-
c.10738C>T	p.Arg3580Trp	rs554959669	0/336	2/2386	0/0/168	0/2/1192	1	1	-
c.10910C>T	p.Ala3637Val	rs751079345	0/336	2/2386	0/0/168	0/2/1192	1	1	-
c.11236A>G	p.Thr3746Ala	-	0/336	1/2387	0/0/168	0/1/1193	1	1	-
c.11367G>A	p.Met3789Ile	-	0/336	1/2387	0/0/168	0/1/1193	1	1	-
c.11512G>C	p.Val3838Leu	rs35332090	20/316	161/2217	1/18/149	5/161/1028	0.49	0.4	0.55
c.11600C>T	p.Thr3867Met	rs199975233	0/336	1/2387	0/0/168	0/1/1193	1	1	-

c.11711G>T	p.Gly3904Val	-	0/336	1/2387	0/0/168	0/1/1193	1	1	-
c.11744A>G	p.Glu3915Gly	rs61740658	20/316	121/2257	1/18/149	5/121/1068	0.7	0.79	0.55
c.11797G>A	p.Val3933Met	rs1181813391	0/336	1/2387	0/0/168	0/1/1193	1	1	-
c.11957G>A	p.Ser3986Asn	rs769487554	0/336	4/2384	0/0/168	0/4/1190	1	1	-
c.11971_11972insAGGG	p.Arg3991Lysfs*67	-	0/336	1/2387	0/0/168	0/1/1193	1	1	-
c.11997C>G	p.Ile3999Met	rs748912687	0/336	1/2387	0/0/168	0/1/1193	1	1	-
c.12179G>A	p.Arg4060His	rs761761703	0/336	1/2387	0/0/168	0/1/1193	1	1	-
c.12198_12199del	p.Asn4066Lysfs*19	-	0/336	1/2387	0/0/168	0/1/1193	1	1	-
c.12362A>G	p.Asn4121Ser	rs143828863	1/335	1/2387	0/1/167	0/1/1193	0.23	0.23	-
c.12527A>G	p.Asn4176Ser	rs527844265	1/335	1/2387	0/1/167	0/1/1193	0.23	0.23	-
c.12577G>C	p.Asp4193His	rs143335048	0/336	2/2386	0/0/168	0/2/1192	1	1	-
c.12698G>A	p.Arg4233His	rs1193186378	0/336	1/2387	0/0/168	0/1/1193	1	1	-
c.12716C>T	p.Ala4239Val	-	2/334	1/2387	0/2/166	0/1/1193	0.04	0.04	-
c.12748C>A	p.Pro4250Thr	rs138029774	1/335	13/2375	0/1/167	0/13/1181	1	1	-
c.12769C>T	p.Gln4257*	rs144160370	0/336	1/2387	0/0/168	0/1/1193	1	1	-
c.12806G>A	p.Arg4269Gln	rs770487709	0/336	1/2387	0/0/168	0/1/1193	1	1	-
c.13084_13085insACCCCCCC	p.Thr4362Asnfs*15	-	0/336	1/2387	0/0/168	0/1/1193	1	1	-
c.13195G>A	p.Ala4399Pro	-	15/321	122/2262	0/0/168	1/0/1193	1	1	1
c.13195G>C	p.Ala4399Thr	rs148731719	0/336	0/2386	0/15/153	2/122/1070	0.6	0.68	1
c.13298G>A	p.Gly4433Glu	rs761606665	0/336	2/2386	0/0/168	0/2/1192	1	1	-

c.13318G>A	p.Asp4440Asn	-	0/336	1/2387	0/0/168	0/1/1193	1	1	-
c.13390C>A	p.Pro4464Thr	-	0/336	1/2387	0/0/168	0/1/1193	1	1	-
c.13699G>A	p.Val4567Met	rs145282452	0/336	1/2387	0/0/168	0/1/1193	1	1	-
c.13726C>T	p.Pro4576Ser	rs776390324	0/336	3/2385	0/0/168	0/3/1191	1	1	-
c.14192G>A	p.Arg4731Gln	rs758730525	0/336	1/2387	0/0/168	0/1/1193	1	1	-
c.14293G>A	p.Val4765Met	-	0/336	2/2386	0/0/168	0/2/1192	1	1	-
c.14429G>A	p.Arg4810Lys	rs112735431	42/294	13/2375	1/40/127	0/13/1181	2.1×10^{-28}*	1.5×10^{-28}*	0.12
c.14842G>A	p.Val4948Met	rs375652626	0/336	1/2387	0/0/168	0/1/1193	1	1	-
c.14997G>C	p.Glu4950Asp	rs371441113	0/336	1/2387	0/0/168	0/1/1193	1	1	-
c.14872C>T	p.Arg4958Trp	rs1048620695	0/336	1/2387	0/0/168	0/1/1193	1	1	-
c.14914A>T	p.Ser4972Cys	-	0/336	1/2387	0/0/168	0/1/1193	1	1	-
c.14923G>A	p.Gly4975Arg	-	0/336	1/2387	0/0/168	0/1/1193	1	1	-
c.15062C>T	p.Ala5021Val	rs138130613	0/336	1/2387	0/0/168	0/1/1193	1	1	-
c.15095_15096insTGG	p.Gly5033dup	-	0/336	1/2387	0/0/168	0/1/1193	1	1	-
c.15130A>C	p.Met5044Leu	-	0/336	1/2387	0/0/168	0/1/1193	1	1	-
c.15254A>G	p.His5085Arg	rs765044536	0/336	2/2386	0/0/168	0/2/1192	1	1	-
c.15458G>A	p.Arg5153His	rs528073196	0/336	1/2387	0/0/168	0/1/1193	1	1	-
c.15575C>T	p.Ser5192Leu	-	1/335	2/2386	0/1/167	0/2/1192	0.33	0.33	-

*Statistically significant (Bonferroni corrected significance level is 8.9×10^{-4} [0.05/56]). Exons and amino acid positions are provided according to the NM_001256071.3 isoform. A: reference allele, a: alternate allele, ICAS: intracranial artery stenosis.

Supplementary Table S2. Nonsynonymous variants of *RNF213* in ICAS patients and control subjects with MAF and the results of in silico analysis

cDNA (NM_001256071.3)	Protein (NP_001243000.2)	rs ID	MAF in ExAC	MAF in 1000 Genomes	MAF in 1000 Genomes (Japanese population)	CADD	SIFT	PolyPhen-2 (HumVar)
c.103G>A	p.Glu35Lys	rs113614776	0.0025	0.0072	0	4.032	tolerated	benign
c.182C>T	p.Pro61Leu	rs9913317	0.0051	0.011	0.015	0.111	tolerated	possibly D*
c.352T>C	p.Cys118Arg	rs201620985	0.0026	0.0002	0	1.792	tolerated	benign
c.355C>T	p.His119Tyr	rs775609914	0.00007	-	-	0.619	tolerated	benign
c.577A>G	p.Ser193Gly	-	-	-	-	0.498	tolerated	benign
c.752_753insT	p.Glu252*	-	-	-	-	NA	NA	NA
c.757C>T	p.Pro253Ser	rs140369116	0.0005	0.0004	0.002	7.935	D*	benign
c.809T>C	p.Met270Thr	rs17857135	0.1592	0.2115	0.065	1.565	tolerated	benign
c.962T>C	p.Met321Thr	rs17853989	0.1797	0.2198	0.066	0.001	tolerated	benign
c.968C>G	p.Ala323Gly	-	-	-	-	0.015	tolerated	benign
c.1035G>C	p.Lys345Asn	-	-	-	-	0.003	tolerated	benign
c.1045G>A	p.Ala349Thr	-	-	-	-	5.531	tolerated	benign
c.1052C>T	p.Ala351Val	rs148593553	0.00005	0.0004	0.002	15.18	tolerated	possibly D*
c.1207A>G	p.Arg403Gly	rs773721359	-	-	-	13.83	D*	possibly D*
c.1271G>A	p.Arg424Lys	-	-	-	-	14.69	tolerated	benign
c.1327C>T	p.His443Tyr	rs199729731	0.00002	0.0002	0.001	0.172	tolerated	benign

c.1762A>G	p.Arg588Gly	rs1390431807	-	-	-	13.83	tolerated	benign
c.2186C>T	p.Pro729Leu	rs72849841	0.124	0.1138	0.015	8.195	tolerated	benign
c.2852A>G	p.His951Arg	rs746475303	0.000009	-	-	11.62	tolerated	benign
c.2867C>T	p.Ser956Leu	rs773701444	0.00002	-	-	14.98	tolerated	benign
c.2936A>G	p.Asn979Ser	rs1049581142	-	-	-	2.096	tolerated	benign
c.3121G>A	p.Ala1041Thr	rs61359568	0.0483	0.0375	0.028	9.729	tolerated	benign
c.3133G>T	p.Asp1045Tyr	-	-	-	-	15.37	NA	possibly D*
c.3286A>G	p.Ile1047Val	-	-	-	-	0.822	tolerated	benign
c.3191G>A	p.Cys1064Tyr	rs1437417566	-	-	-	0.526	tolerated	benign
c.3212T>C	p.Leu1071Pro	-	-	-	-	15.03	D*	possibly D*
c.3397C>A	p.Gln1133*	-	-	-	-	24.8	NA	NA
c.3397C>T	p.Gln1133Lys	rs8082521	0.3638	0.4417	0.226	0.002	tolerated	benign
c.3512T>C	p.Ile1171Thr	-	-	-	-	13.69	D*	benign
c.3583G>A	p.Val1195Met	rs10782008	0.4236	0.4888	0.388	6.93	tolerated	benign
c.3701A>G	p.Gln1234Arg	rs116722283	0.0104	0.0323	0	17.13	tolerated	benign
c.3814G>C	p.Glu1272Gln	rs9913636	0.4389	0.3433	0.355	5.478	tolerated	possibly D*
c.3992A>G	p.Asp1331Gly	rs8074015	0.356	0.4034	0.395	2.698	tolerated	benign
c.3992A>T	p.Asp1331Val	-	0.356	0.4034	0.395	2.646	tolerated	benign
c.4031A>G	p.Lys1344Arg	-	-	-	-	2.164	tolerated	benign
c.4274A>G	p.Lys1425Arg	-	-	-	-	7.741	tolerated	benign
c.4316A>G	p.Asn1439Ser	rs761802191	0.00006	-	-	15.39	tolerated	benign
c.4336G>C	p.Asp1446His	-	-	-	-	25.5	D*	probably D*

c.4417G>A	p.Ala1473Thr	rs1428500978	-	-	-	14.96	D*	possibly D*
c.4626A>C	p.Gln1542His	-	-	-	-	1.408	D*	benign
c.4691A>G	p.His1564Arg	rs1568090106	-	-	-	0.081	tolerated	benign
c.4865C>A	p.Ala1622Asp	rs534665632	0.0001	0.0002	0.001	16.07	tolerated	benign
c.5132A>G	p.Tyr1711Cys	-	-	-	-	22	D*	probably D*
c.5221G>A	p.Val1741Ile	rs1392164744	-	-	-	8.044	tolerated	benign
c.5267G>A	p.Arg1756His	rs772938055	0.00006	-	-	0.012	tolerated	benign
c.5449G>T	p.Val1817Leu	-	-	-	-	10.08	tolerated	possibly D*
c.5577C>G	p.Tyr1859*	-	-	-	-	34	NA	NA
c.5597C>T	p.Thr1866Ile	rs546687179	0.00007	0.0004	0.002	9.769	D*	probably D*
c.5795G>A	p.Gly1932Glu	-	-	-	-	2.361	tolerated	benign
c.5909C>T	p.Pro1970Leu	rs768990872	0.000008	-	-	18.56	D*	possibly D*
c.5972C>T	p.Ser1991Leu	rs1164127473	-	-	-	24.4	D*	probably D*
c.6019G>A	p.Asp2007Asn	rs369925564	0.00005	0.0002	0.001	3.83	tolerated	benign
c.6034C>T	p.Gln2012*	-	-	-	-	34	NA	NA
c.6202G>A	p.Val2068Met	-	-	-	-	17	tolerated	benign
c.6320C>T	p.Pro2107Leu	rs756213942	0.000008	-	-	1.612	tolerated	possibly D*
c.6575G>A	p.Gly2192Asp	rs755712045	0.00002	-	-	19.98	D*	probably D*
c.6611A>G	p.His2204Arg	rs774152938	0.00002	-	-	20.6	D*	benign
c.6659G>A	p.Arg2220Gln	rs764839860	0.00006	-	-	12.43	tolerated	benign
c.6802C>T	p.His2268Tyr	rs764823009	0.000008	-	-	3.347	tolerated	benign
c.6979A>G	p.Asn2327Asp	rs138044665	0.001	0.0016	0	4.35	tolerated	benign

c.7001G>A	p.Ser2334Asn	rs9674961	0.3919	0.4415	0.376	5.992	tolerated	benign
c.7066C>T	p.Leu2356Phe	rs200724769	0.00002	-	-	14.16	D*	benign
c.7232G>A	p.Arg2411Gln	rs768216512	0.00002	-	-	25.1	D*	probably D*
c.7250T>G	p.Ile2417Ser	rs181965032	0.0007	0.0014	0.007	25.1	D*	probably D*
c.7319G>A	p.Gly2440Asp	rs761027115	0.00002	-	-	13.64	tolerated	benign
c.7348A>G	p.Lys2450Glu	rs1299071699	-	-	-	23.7	D*	probably D*
c.7662C>A	p.Asp2554Glu	rs138516230	0.0004	0.001	0.005	5.695	tolerated	benign
c.8020A>G	p.Asn2674Asp	-	-	-	-	0.117	tolerated	benign
c.8217G>T	p.Arg2739Ser	-	-	-	-	16.92	tolerated	possibly D*
c.8389C>A	p.Arg2797Ser	rs776731965	0.00002	-	-	23.8	tolerated	possibly D*
c.8983G>A	p.Ala2995Thr	rs374068975	0.00004	-	-	13.5	tolerated	benign
c.9013G>A	p.Glu3005Lys	rs147076172	0.00002	0.0002	0.001	17.44	tolerated	possibly D*
c.9022T>C	p.Cys3008Arg	rs61600413	0.055	0.1038	0.009	5.608	tolerated	benign
c.9245A>G	p.Gln3082Arg	-	-	-	-	24.2	D*	probably D*
c.9404C>T	p.Thr3135Ile	-	-	-	-	24.1	D*	probably D*
c.9422G>A	p.Arg3141Gln	-	-	-	-	23.1	D*	possibly D*
c.9439C>T	p.Arg3147Cys	rs781176178	0.000008	-	-	25.4	D*	probably D*
c.9542A>G	p.Glu3181Gly	-	-	-	-	16.72	D*	possibly D*
c.9679G>C	p.Ala3227Pro	rs776295572	0.000008	-	-	22.8	D*	probably D*
c.9767C>T	p.Ser3256Leu	rs529113475	0.0002	0.0002	0.001	5.622	tolerated	benign
c.9806G>C	p.Arg3269Pro	rs377303933	0.00002	-	-	23.2	D*	probably D*
c.9909C>A	p.His3303Gln	-	-	-	-	2.09	D*	benign

c.9985G>T	p.Asp3329Tyr	-	-	-	-	24.6	D*	probably D*
c.10403C>T	p.Ala3468Val	rs142798005	0.0002	0.0006	0.002	0.062	tolerated	benign
c.10617G>C	p.Glu3490Asp	rs7216493	0.3343	0.371	0.549	1.031	D*	possibly D*
c.10491_10492insGGA GGTGGCAGAGG	p.Glu3498Glyfs*44	-	-	-	-	NA	NA	NA
c.10510G>A	p.Glu3504Lys	rs751952931	0.00002	-	-	14.71	tolerated	benign
c.10526_10527insCA	p.Glu3510Lysfs*28	-	-	-	-	NA	NA	NA
c.10532G>A	p.Ser3511Asn	rs749427375	0.00005	-	-	7.207	tolerated	benign
c.10579G>A	p.Val3527Met	rs547259103	0.00002	0.0002	0.001	1.074	tolerated	benign
c.10663A>G	p.Thr3555Ala	-	-	-	-	2.128	tolerated	benign
c.10675C>T	p.Arg3559Trp	rs368365560	0.000008	-	-	12.6	D*	probably D*
c.10684G>C	p.Val3562Leu	-	-	-	-	0.111	tolerated	benign
c.10727-2A>G	-	-	-	-	-	NA	NA	NA
c.10738C>T	p.Arg3580Trp	rs554959669	0.0001	0.0002	0.001	14.61	tolerated	benign
c.10910C>T	p.Ala3637Val	rs751079345	0.000008	-	-	23.5	tolerated	probably D*
c.11236A>G	p.Thr3746Ala	-	-	-	-	22.5	D*	possibly D*
c.11367G>A	p.Met3789Ile	-	-	-	-	14.19	tolerated	benign
c.11512G>C	p.Val3838Leu	rs35332090	0.1089	0.1462	0.042	1.011	tolerated	benign
c.11600C>T	p.Thr3867Met	rs199975233	0.0002	-	-	6.311	tolerated	benign
c.11711G>T	p.Gly3904Val	-	-	-	-	9.377	tolerated	benign
c.11744A>G	p.Glu3915Gly	rs61740658	0.0848	0.0811	0.037	13.48	D*	benign
c.11797G>A	p.Val3933Met	rs1181813391	-	-	-	23.9	tolerated	possibly D*

c.11957G>A	p.Ser3986Asn	rs769487554	0.000008	-	-	13.32	tolerated	benign
c.11971_11972insAGGG	p.Arg3991Lysfs*67	-	-	-	-	NA	NA	NA
c.11997C>G	p.Ile3999Met	rs748912687	0.000008	-	-	15.24	D*	probably D*
c.12179G>A	p.Arg4060His	rs761761703	0.000008	-	-	17.7	tolerated	benign
c.12198_12199del	p.Asn4066Lysfs*19	-	0.000008	-	-	NA	NA	NA
c.12362A>G	p.Asn4121Ser	rs143828863	0.0001	-	-	19.39	tolerated	benign
c.12527A>G	p.Asn4176Ser	rs527844265	0.0001	0.0002	0.001	13.44	tolerated	benign
c.12577G>C	p.Asp4193His	rs143335048	0.000009	0.0002	0.001	11.57	tolerated	benign
c.12698G>A	p.Arg4233His	rs1193186378	-	-	-	25	D*	probably D*
c.12716C>T	p.Ala4239Val	-	-	-	-	18.2	tolerated	probably D*
c.12748C>A	p.Pro4250Thr	rs138029774	0.0061	0.0046	0.005	1.082	tolerated	benign
c.12769C>T	p.Gln4257*	rs144160370	-	-	-	36	NA	NA
c.12806G>A	p.Arg4269Gln	rs770487709	0.00002	-	-	0.134	tolerated	benign
c.13084_13085insACCCCCCC	p.Thr4362Asnfs*15	-	-	-	-	NA	NA	NA
c.13195G>A	p.Ala4399Pro	-	0.0093	0.0182	0.068	16.67	D*	benign
c.13195G>C	p.Ala4399Thr	rs148731719	0.0093	0.0182	0.068	13.67	tolerated	probably D*
c.13298G>A	p.Gly4433Glu	rs761606665	0.00002	-	-	8.57	tolerated	benign
c.13318G>A	p.Asp4440Asn	-	-	-	-	1.375	tolerated	benign
c.13390C>A	p.Pro4464Thr	-	-	-	-	24.3	D*	probably D*
c.13699G>A	p.Val4567Met	rs145282452	0.0001	0.0006	0	8.221	tolerated	benign

c.13726C>T	p.Pro4576Ser	rs776390324	0.00002	-	-	23.2	D*	probably D*
c.14192G>A	p.Arg4731Gln	rs758730525	0.00002	-	-	0.153	tolerated	benign
c.14293G>A	p.Val4765Met	-	-	-	-	23.4	D*	probably D*
c.14429G>A	p.Arg4810Lys	rs112735431	0.0004	0.0012	0.002	12.8	Tolerated	benign
c.14842G>A	p.Val4948Met	rs375652626	0.00007	-	-	0.099	Tolerated	benign
c.14997G>C	p.Glu4950Asp	rs371441113	0.0003	0.0008	0.004	17.73	Tolerated	possibly D*
c.14872C>T	p.Arg4958Trp	rs1048620695	-	-	-	24.1	D*	probably D*
c.14914A>T	p.Ser4972Cys	-	-	-	-	17.51	D*	possibly D*
c.14923G>A	p.Gly4975Arg	-	-	-	-	29.1	D*	possibly D*
c.15062C>T	p.Ala5021Val	rs138130613	0.0003	0.0008	0.004	16.46	Tolerated	benign
c.15095_15096insTGG	p.Gly5033dup	-	-	-	-	NA	NA	NA
c.15130A>C	p.Met5044Leu	-	-	-	-	15.08	Tolerated	benign
c.15254A>G	p.His5085Arg	rs765044536	0.00006	-	-	23	Tolerated	benign
c.15458G>A	p.Arg5153His	rs528073196	0.00003	0.0002	0.001	14.79	Tolerated	possibly D*
c.15575C>T	p.Ser5192Leu	-	-	-	-	11.17	Tolerated	benign

Exons and amino acid positions are provided according to the NM_001256071.3 isoform. CADD: Combined Annotation Dependent Depletion, D*: damaging, ExAC: Exome Aggregation Consortium, 1000 Genomes: 1000 Genomes project, MAF: minor allele frequency, NA: not available, -: novel variant.

Supplementary Table S3. Results of burden tests for nonsynonymous variants detected in our subjects without p.Arg4810Lys

	**N-variants in burden test**	**Cases (without p.Arg4810Lys) with at least one variant (total 127)**	**Controls (without p.Arg4810Lys) with at least one variant (total 1181)**	**OR (95% CI)**	**P Value**
Any-MAF	49				
CADD >0	49	127	1,176	-	0.99
CADD >10	20	127	1,176	-	0.99
CADD >20	5	2	49	0.39 (0.09-1.62)	0.19
MAF <0.01	35				
CADD >0	35	40	369	1.03 (0.69-1.53)	0.89
CADD >10	17	22	207	1.00 (0.61-1.62)	0.98
CADD >20	5	2	44	0.43 (0.10-1.78)	0.24
MAF <0.001	30				
CADD >0	30	16	197	0.76 (0.44-1.31)	0.33
CADD >10	16	9	88	1.01 (0.49-2.06)	0.98
CADD >20	5	2	44	0.43 (0.10-1.78)	0.24
MAF <0.0001	24				
CADD >0	24	11	123	0.87 (0.45-1.66)	0.67
CADD >10	15	8	80	0.97 (0.46-2.07)	0.94
CADD >20	5	2	43	0.44 (0.11-1.85)	0.26
MAF <0.00001	18				
CADD >0	18	11	83	1.07 (0.52-2.20)	0.85
CADD >10	13	8	60	1.17 (0.52-2.64)	0.70
CADD >20	5	2	33	0.59 (0.14-2.50)	0.47

*Statistically significant (Bonferroni corrected significance level is 3.3×10^{-3} [0.05/15]). CI: confidence interval, OR: odds ratio.

Supplementary Table S4. Previously reported *RNF213* rare variants in MMD patients described by protein change

Protein (NP_001243000.2)	Ethnicity	Study
**Asian**		
p.Gly517Arg	Chinese	Shoemaker et al.
p.Pro800Leu	Japanese	Miyatake et al.
p.Glu996Lys	Japanese	Akagawa et al.
p.Ala1041Val	Chinese	Zhang et al.
p.Arg1228Gly	Chinese	Zhang et al.
p.Ser1474Phe	Filipino	Shoemaker et al.
p.Ala1622Val	Taiwanese	Lee et al.
p.Thr1705Lys	Japanese	Miyatake et al.
p.Glu1707Lys	Chinese	Zhang et al.
p.Thr1727Met	Chinese	Zhang et al.
p.Thr1866Ile	Chinese, Japanese	Miyatake et al., Zhang et al.
p.Leu1911Ile	Japanese	Miyatake et al.
p.Arg2089Trp	Japanese	Miyatake et al.
p.Pro2107Leu	Chinese	Zhang et al.
p.Ile2240Thr	Sri Lankan	Shoemaker et al.
p.Leu2356Phe	Japanese	Miyatake et al.
p.Ile2417Ser	Japanese	Miyatake et al.
p.Asp2554Glu	Japanese, Korean	Shoemaker et al.
p.Asn2971Tyr	Chinese	Zhang et al.
p.Gln3020Leu	Japanese	Moteki et al.
p.Glu3061Lys	Chinese	Zhang et al.
p.Gln3082Arg	Japanese	Miyatake et al.
p.Ser3256Leu	Japanese	Miyatake et al.
p.Thr3316Ile	Japanese	Moteki et al.
p.Ala3468Val	Japanese	Moteki et al.
p.Gly3470Arg	Chinese	Zhang et al.
p.Arg3580Trp	Chinese	Zhang et al.
p.Met3666Thr	Chinese	Shoemaker et al., Zhang et al.
p.Ile3693Leu	Chinese	Zhang et al.

p.Met3891Val	Japanese	Kamada et al.
p.Asp3899Asn	Chinese	Zhang et al.
p.Val3933Met	Taiwanese	Lee et al.
p.Gly3936Glu	Chinese	Zhang et al.
p.Lys3982Arg	Chinese	Shoemaker et al.
p.Pro4007Arg	Chinese	Wu et al.
p.Asp4013Asn	Chinese	Zhang et al.
p.His4014Thr	Chinese	Zhang et al.
p.Trp4024Arg	Japanese	Miyatake et al.
p.Ala4050Val	Chinese	Zhang et al.
p.His4058Pro	Japanese	Akagawa et al.
p.Arg4062Gln	Chinese, Japanese	Moteki et al., Zhang et al., Akagawa et al.
p.Asn4066Ser	Chinese	Zhang et al.
p.Ile4076Val	Japanese/Filipino	Cecchi et al.
p.Arg4131Cys	Taiwanese	Lee et al.
p.Lys4160Gln	Chinese	Zhang et al.
p.Asp4180Asn	Sri Lankan	Shoemaker et al.
p.Met4289Ile	Chinese	Zhang et al.
p.Gln4367Leu	Chinese	Wu et al.
p.Ser4389Gly	Chinese	Zhang et al.
p.Val4567Met	Japanese	Kamada et al.
p.Pro4576Ser	Chinese	Zhang et al.
p.Thr4586Pro	Chinese	Wu et al.
p.Leu4631Val	Chinese	Wu et al.
p.Asp4636Asn	Japanese	Miyatake et al.
p.Gly4640Arg	Chinese	Zhang et al.
p.Glu4750Lys	Japanese	Miyatake et al.
p.Val4765Met	Japanese	Kamada et al.
p.Arg4810Lys	Bangladeshi, Chinese, Indian, Japanese, Korean, Taiwanese	Cecchi et al., Kamada et al., Lee et al., Liu et al., Miyatake et al., Moteki et al., Wu et al., Zhang et al.
p.Arg4810Gly	Filipino	Shoemaker et al.
p.Asp4863Asn	Chinese	Liu et al., Zhang et al.

p.Val4884Ile	Chinese	Zhang et al.
p.Glu4917Lys	Japanese	Miyatake et al.
p.Arg4927Gln	Japanese	Miyatake et al.
p.Gly4938Arg	Chinese	Zhang et al.
p.Glu4950Asp	Chinese	Liu et al., Wu et al., Zhang et al.
p.Ser5012Arg	Chinese	Zhang et al.
p.Ala5021Val	Chinese	Liu et al., Wu et al., Zhang et al.
p.Thr5037Ile	Chinese	Zhang et al.
p.Met5136Ile	Chinese	Wu et al.
p.Arg5153His	Chinese	Zhang et al.
p.Asp5160Glu	Chinese	Liu et al.
p.Glu5176Gly	Chinese	Liu et al.
**Non-Asian**		
p.Glu35Lys	African American	Shoemaker et al.
p.Cys118Arg	Caucasian	Guey et al.
p.Leu133Met	Caucasian	Shoemaker et al., Guey et al.
p.Ile209Asn	Caucasian	Shoemaker et al., Guey et al.
p.Gln239Glu	Caucasian	Shoemaker et al.
p.Pro395Leu	Caucasian	Guey et al.
p.Ala529del	European American	Cecchi et al.
p.Pro1036Leu	Caucasian	Shoemaker et al.
p.Ala1135Val	Caucasian	Guey et al.
p.Gln1470Arg	Caucasian	Raso et al.
p.Gln1549Lys	Caucasian	Shoemaker et al.
p.Thr1705Lys	Caucasian	Guey et al.
p.Pro1721Leu	Caucasian	Guey et al., Raso et al.
p.Ala1844Thr	Caucasian	Guey et al.
p.His3160Arg	African American	Shoemaker et al.
p.Ile3318Val	Caucasian	Raso et al.
p.Arg3559Gln	Caucasian	Shoemaker et al.
p.Met3699Thr	Caucasian	Shoemaker et al.
p.Arg3846His	Caucasian	Guey et al.
p.Arg3922Gln	European American	Cecchi et al.
p.Ala3927Thr	Caucasian	Guey et al., Shoemaker et al.

p.Val3933Met	Caucasian	Guey et al.
p.Val3941Ile	German	Liu et al.
p.Asn3962Asp	European American	Cecchi et al.
p.Cys3997Tyr	European American	Cecchi et al.
p.Asp4013Asn	Caucasian, European American, Slovakian/Czech	Cecchi et al., Guey et al., Kobayashi et al., Liu et al.
p.His4014Asn	Caucasian	Guey et al.
p.Cys4017Ser	Caucasian	Raso et al.
p.Arg4019Cys	Caucasian, European American, Hispanic, Slovakian/Czech	Cecchi et al., Guey et al., Kobayashi et al., Shoemaker et al.
p.Met4030Thr	Caucasian	Shoemaker et al.
p.Cys4032Arg	Caucasian	Guey et al.
p.Pro4033Leu	Caucasian	Guey et al.
p.Glu4042Lys	Caucasian, Slovakian/Czech	Kobayashi et al., Guey et al.
p.His4051Pro	Caucasian	Guey et al.
p.Arg4062Gln	Caucasian, German, Hispanic	Guey et al., Liu et al., Shoemaker et al.
p.Lys4115del	European American	Cecchi et al.
p.Ser4118Phe	Caucasian	Harel et al.
p.Asp4122Val	Caucasian	Guey et al.
p.Val4146Ala	Slovakian/Czech	Kobayashi et al.
p.Asp4237Glu	Hispanic	Cecchi et al.
p.Asp4273Asn	Caucasian, Hispanic	Shoemaker et al.
p.Val4280Leu	Caucasian	Shoemaker et al.
p.Val4453Ile	Caucasian, African American	Shoemaker et al.
p.Pro4608Ser	Caucasian, German	Liu et al., Guey et al.
p.Gly4640Ser	Caucasian	Guey et al.
p.Lys4732Thr	Hispanic	Cecchi et al.
p.Val4804Leu	African American	Shoemaker et al.
p.Glu4940-Phe4950ins7	European American	Cecchi et al.
p.Val5163Ile	European American	Cecchi et al.

Exons and amino acid positions are provided according to the NP_001243000.2 isoform.
